# Rehabilitation of Maxillary Defect Using Zygomatic Implant Retained Obturator

**DOI:** 10.1155/2021/2391331

**Published:** 2021-10-13

**Authors:** Mounika Ayinala, Gautam Shetty

**Affiliations:** Department of Prosthodontics, Rajarajeswari Dental College and Hospital, Bengaluru, Karnataka, India

## Abstract

Tumors involving the hard palate, maxillary sinus, or nasal cavity require maxillectomy based on the extent of the lesion. Lack of these boundaries affects the speech, esthetics, and masticatory function. Prosthetic rehabilitation of these defects can be done utilizing zygomatic implants. This present case describes the use of a zygomatic implant to retain a maxillary obturator in a 22-year-old male patient following partial maxillectomy (Brown's Class 2b) due to odontogenic myxoma. A surgical obturator was secured in position subsequent to the implant placement. Following the healing period, an interim obturator using heat cure acrylic was fabricated. Mechanical retention for the definitive obturator was obtained through the ball attachment suspended from the multiunit abutment of the zygomatic implant. The case was followed up closely for a year to evaluate the function of the prosthesis. The prosthetic rehabilitation not only promoted esthetics and function but also improved the patient's quality of life.

## 1. Introduction

A challenging endeavor for prosthodontists is rehabilitating patients with maxillectomy/atrophic maxilla as it is a complex area. Carcinomas involving the hard palate, maxillary sinus, and nasal cavity will require maxillectomy, causing dysfunction of the stomatognathic system and thus affecting the patients' quality of life [1]. Tumors such as odontogenic myxoma are uncommon and have the potential for destruction of the jaws. They arise from the dental papilla, dental follicle, or periodontal ligament and occur during the second/third decade of life. These tumors are painless, slow-growing, and invasive with the expansion of bony cortices, causing asymmetry of the face [2, 3]. Management of these carcinomas is through a surgical approach which ranges from enucleation with curettage, segmental resection to hemimaxillectomy depending on the size, and extent of the lesion. They cannot be managed through radiotherapy and chemotherapy as they are not radiosensitive [4, 5].

A custom-made dental prosthesis such as an obturator plays an important role to promote function and esthetics and helps in maintaining the oro-nasal separation. Obtaining retention and stability depends on the extent of the defect, the remaining number of natural teeth, the amount of residual bone, and the patients' ability to adapt to the prosthesis [6]. Usually, because of limited bone support and large defects, the function of an obturator is compromised. But, with the advent of osseointegrated implants, better retention and support can be derived for the implant-retained obturator with less discomfort, complications, and time [7]. Apart from endosseous implants, zygomatic implants have gained popularity over recent years. Branemark introduced it in 1998 for its use in the atrophic maxilla, tumor resection defects, and congenital defects. Later, in the year 2001, Branemark and his team published the first paper on the survival of zygomatic implants placed to obtain prosthetic anchorage in patients with maxillary defects [8]. The 12-year cumulative survival rate of these implants is 95.2% and can be used in rehabilitation after tumor resection/trauma/atrophic maxilla without hard and soft tissue augmentations [9].

This clinical report describes the successful management of a patient with maxillary odontogenic myxoma who underwent partial maxillary resection followed by prosthesis using a zygomatic implant.

## 2. Case Report

**Figure 1:**
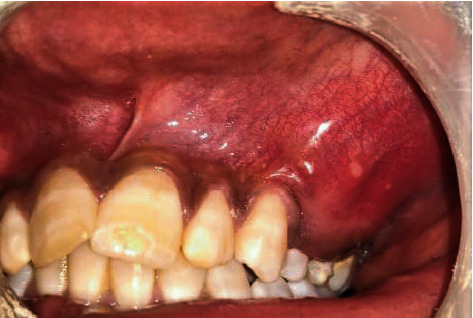
Intraoral view of the lesion.

A 22-year-old male patient was referred to Rajarajeswari Dental College and Hospital with a complaint of swelling in the left maxillary posterior region. On extraoral examination, no gross facial asymmetry was noticed. Intraorally, there was no pain/mobility of the teeth. The swelling extended from 23 to 26 region as shown in Figure 1. Radiographic and histopathological examinations confirmed the diagnosis, i.e., odontogenic myxoma involving the left maxilla. A partial left-sided maxillectomy was planned, and the patient was referred to the department of prosthodontics for the fabrication of a surgical obturator. A preoperative impression was made using irreversible hydrocolloid, and the casts were poured using dental stone. The area of resection was delineated by the surgeon and accordingly, the cast was scrapped to fabricate a surgical obturator.

**Figure 2:**
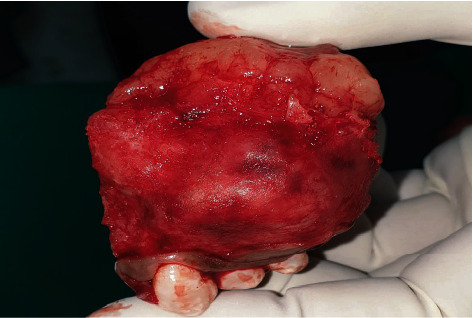
Excised tissues.

**Figure 3:**
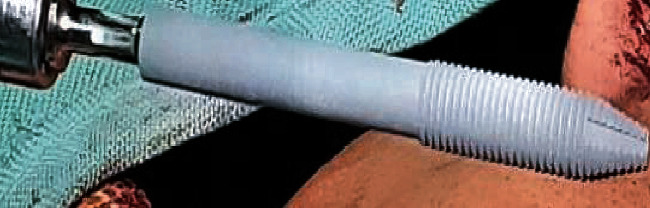
Zygomatic implant.

Through the intraoral approach, partial maxillectomy was carried out by preserving the frontal wall of the maxillary antrum, zygoma, and the floor of the orbit. The anterior cut was undertaken through the left lateral incisor and limiting the posterior cut up to the 1st molar as seen in Figure 2. The histopathological report revealed that the tissue was excised with good margins and did not require any adjunctive therapy. A 42.5 mm zygomatic implant (NobelZygoma, Nobel BioCare, United States) as seen in Figure 3 was placed in the left zygomatic bone with maximum stability, ensuring the retention of the obturator through proper positioning of the prosthetic head beneath the body of the prosthesis. The buccal and palatal flaps were saved and sutured before the placement of a surgical obturator which was screwed in with the help of a multiunit abutment and a prosthetic screw. The surgical obturator was cleaned every now and then until an interim obturator was fabricated.

**Figure 4:**
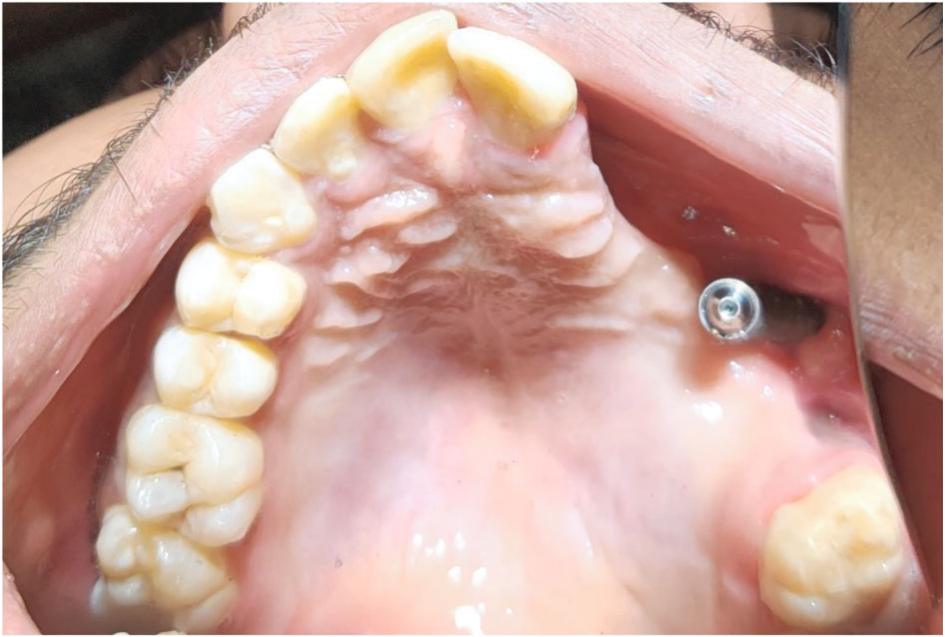
Intraoral view after 5 weeks of healing period.

After 4 weeks of the healing period, the patient was recalled for the fabrication of an interim obturator. There was no evidence of infection with good healing of the soft tissues around the abutment as seen in Figure 4. A removable interim obturator was fabricated conventionally using heat cure acrylic resin by making the preliminary impressions. Jaw relation and teeth arrangement were done by relieving the abutment area. The interim obturator was fabricated using compression molding technique, and retention was obtained by placing retainers on the central incisor, molar, and premolars of the contralateral arch.

**Figure 5:**
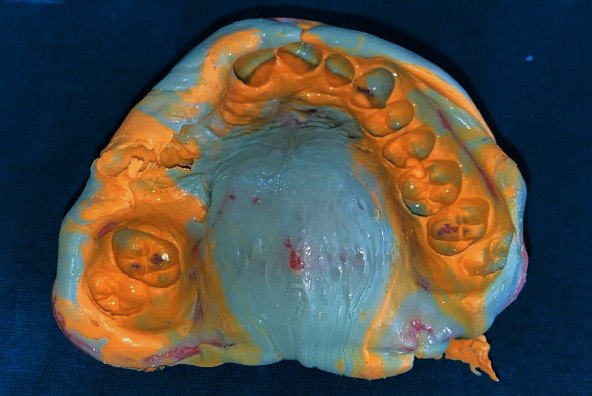
Definitive impression following mouth preparation.

**Figure 6:**
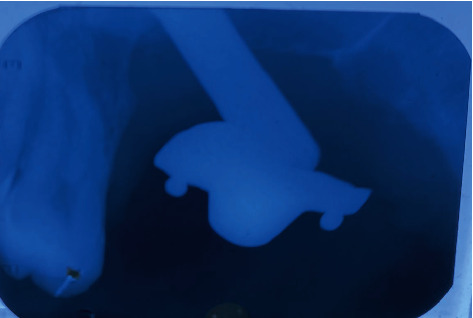
IOPA of O-ball attachment secured with prosthetic screw over multiunit abutment.

**Figure 7:**
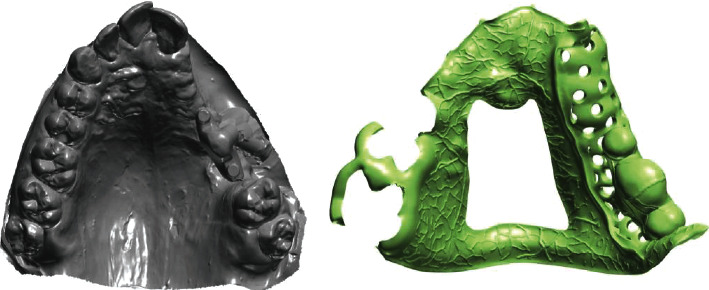
.STL file of the scanned cast and designing of cast partial denture.

**Figure 8:**
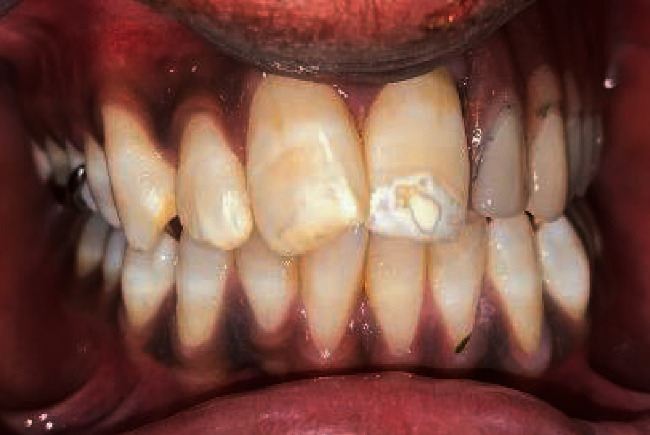
Final prosthesis.

Five months following the surgery, a fixed-removable prosthesis was planned by utilizing precision attachments. The diagnostic casts were mounted on a semiadjustable articulator following a face bow transfer, and the amount of space available for the attachment was evaluated using a putty index. As the available space was more than required (15 mm), a ball and socket attachment system was planned with a cast partial denture. Occlusal rest seats were prepared on 15 and 16 to receive an embrasure clasp, and the guide planes were prepared on 21 and 27, respectively. Following mouth preparation, a custom tray was fabricated to make an abutment level impression using heavy and light body consistency of polyvinyl siloxane impression material as seen in Figure 5. The accuracy of the definitive cast was verified using a jig trail. Two microballs of 2.5 mm diameter (Rhein 83, NY) were casted and secured using a prosthetic screw on the multiunit abutment, and the fit was confirmed using an IOPA as seen in Figure 6. The definitive cast with ball attachment was scanned using an optical scanner, converted to .STL (Standard Tessellation Language) format for computer-aided designing of cast partial denture as seen in Figure 7. A selective laser melting of cobalt-chromium was employed for the fabrication of the denture, and the trail fit was verified. Following jaw relation, the esthetics and speech were verified with the trail denture in place. Chair-side pickup of the final denture was done to incorporate the metal housings with nylon caps for retention. The patient was educated about oral hygiene and how to handle the prosthesis. The clinical outcome included improvement of esthetics; function and the sense of confidence and security were restored in the patient as seen in Figure 8. A twelve-month follow-up did not show any evidence of infection, and the patient was asked to return for check-ups at 6-month interval.

## 3. Discussion

Various procedures such as obturators, local flaps, and microvascular flaps have been advocated to reconstruct the maxilla following the surgical resection [10–13]. Microvascular reconstruction poses certain clinical risks as there could be donor site morbidity, failure of flap or fibrous union, etc. [14]. Also, the retention and stability factors of the conventional obturators are compromised in extensive maxillary defects. The supporting bone plays an important role in the success of a prosthesis, and hence, it is easier to restore subjects with partial/hemi maxillectomy compared to total maxillectomy.

The amount of remaining horizontal component is the deciding factor in restoring the subjects with an obturator. A significant amount of forces can be distributed on the remaining dentition through clasps to retain the prosthesis in position [15]. Due to the limited availability of the bone, the success of endosteal implants in the long term is questionable. In the present case, since the hard palate was sectioned, the prosthetic rehabilitation was planned with an obturator supported by a zygomatic implant considering the age of the patient. The major advantage of these implants is that they eliminate the use of graft material and its associated infections [16, 17].

Apart from this, there are certain complications associated with the placement of zygomatic implants, such as orbital injury/penetration, oroantral fistula formation, sensory nerve deficits temporarily, and vestibular cortical fenestration [18]. To overcome these complications, it is important to plan the placement of the implant based on a CT/CBCT imaging, and the skill of the surgeon plays a crucial role. As mentioned previously, there were no postoperative complications in the present case except for the sensitivity of soft tissue around the implant during the 12-month follow-up period, but this did not limit the functionality of the prosthesis.

## 4. Conclusion

Zygomatic implants have proven to be an alternative to the traditional methods in restoring subjects with maxillectomy defects as they help in retaining the obturators. With a proper diagnosis and treatment plan, a multidisciplinary approach involving a radiologist, surgeon, and prosthodontist helps to reconstruct and rehabilitate at various stages of treatment. The ultimate goal of the clinician is to improve the quality of life of the patient.

## References

[1] Taylor T. D. (2000). *Clinical Maxillofacial Prosthetics*.

[2] Shafer W., Hine M., Levy B. (1983). *A Textbook of Oral Pathology*.

[3] Kramer I., Pindborg J., Shear M. (1992). *Histological Typing of Odontogenic Tumours*.

[4] Barros R. E., Dominguez F. V., Cabrini R. L. (1969). Myxoma of the jaws. *Oral Surgery*.

[5] Deron P. B., Nikolovski N., den Hollander J. C., Spoelstra H. A., Knegt P. P. (1996). Myxoma of the maxilla: a case with extremely aggressive biologic behavior. *Head & Neck*.

[6] Koyama S., Sasaki K., Inai T., Watanabe M. (2005). Effects of defect configuration, size, and remaining teeth on masticatory function in post-maxillectomy patients. *Journal of Oral Rehabilitation*.

[7] Roumanas E., Nishimura R. D., Davis B. K. (1997). Clinical evaluation of implants retaining edentulous maxillary obturator prostheses. *The Journal of Prosthetic Dentistry*.

[8] Parel S. M., Brånemark P. I., Ohrnell L. O., Svensson B. (2001). Remote implant anchorage for the rehabilitation of maxillary defects. *The Journal of Prosthetic Dentistry*.

[9] Chrcanovic B. R., Albrektsson T., Wennerberg A. (2016). Survival and complications of zygomatic implants: an updated systematic review. *Journal of Oral and Maxillofacial Surgery*.

[10] Kornblith A. B., Zlotolow I. M., Gooen J. (1996). Quality of life of maxillectomy patients using an obturator prosthesis. *Head & Neck*.

[11] Cordeiro P. G., Santamaria E. (2000). A classification system and algorithm for reconstruction of maxillectomy and midfacial defects. *Plastic and Reconstructive Surgery*.

[12] Mücke T., Hölzle F., Loeffelbein D. J. (2011). Maxillary reconstruction using microvascular free flaps. *Oral Surgery, Oral Medicine, Oral Pathology, Oral Radiology, and Endodontics*.

[13] Sakuraba M., Kimata Y., Ota Y. (2003). Simple maxillary reconstruction using free tissue transfer and prostheses. *Plastic and Reconstructive Surgery*.

[14] Dattani A., Richardson D., Butterworth C. J. (2017). A novel report on the use of an oncology zygomatic implant-retained maxillary obturator in a paediatric patient. *International Journal of Implant Dentistry*.

[15] Okay D. J., Genden E., Buchbinder D., Urken M. (2001). Prosthodontic guidelines for surgical reconstruction of the maxilla: a classification system of defects. *The Journal of Prosthetic Dentistry*.

[16] Mittal S., Agarwal M. (2018). *Case Reports in Dentistry*.

[17] Agbara R., Goetze E., Koch F., Wagner W. (2017). Zygoma implants in oral rehabilitation: a review of 28 cases. *Dental Research Journal*.

[18] Tzerbos F., Bountaniotis F., Theologie-Lygidakis N., Fakitsas D., Fakitsas I. (2016). Complications of zygomatic implants: our clinical experience with 4 cases. *Acta Stomatologica Croatica*.

